# *Salmonella* Enteritidis reduction in layer ceca with a *Bacillus* probiotic

**DOI:** 10.14202/vetworld.2020.184-187

**Published:** 2020-01-25

**Authors:** Paul T. Price, Thomas A. Gaydos, Roy D. Berghaus, Virginia Baxter, Charles L. Hofacre, Michael D. Sims

**Affiliations:** 1 Phileo by Lesaffre Milwaukee, Wisconsin, United States; 2 Department of Food, Nutrition, and Packaging Sciences Clemson University Clemson, South Carolina, United States; 3 Gaydos Technical Services, LLC, Dallas, Texas, United States; 4 Department of Population Health College of Veterinary Medicine, University of Georgia, Athens, Georgia; 5 Southern Poultry Research Group, Inc., Watkinsville, Georgia; 6 Virginia Diversified Research, Harrisonburg, Virginia, United States

**Keywords:** layers, probiotic, *Salmonella* Enteritidis, yeast cell wall, yeast culture

## Abstract

**Background and Aim:**

*Salmonella* Enteritidis (SE) is a significant foodborne pathogen that can often be traced to poultry and poultry products. This study aims to evaluate the ability of three commonly used non-antimicrobial feed additives in reducing the amount of SE in the ceca of laying type pullets.

**Materials and Methods:**

On day 0, 60 Hy-Line Brown pullets aged 9 weeks were allocated to individual cages in 15 replicate blocks of four pens. Pullets were administered a mash feed provided *ad libitum* without supplementation (control) or with dietary supplementation of 454 g/ton yeast cell wall (YCW), or 454 g/ton *Bacillus* spp. probiotic, or 1133 g/ton yeast culture (YC). On day 3 of the trial, all birds were orally administered 3×10^7^ CFU of a nalidixic acid-resistant SE. On day 10, 7 days after inoculation, all birds were humanely euthanized, and the ceca were aseptically removed for analysis.

**Results:**

There was no significant difference in the prevalence of SE among treatments. The mean quantity of SE detected in the ceca expressed in log_10_ most probable number/g was 2.52 in the control, 2.49 in the YCW treatment, 1.73 in the probiotic treatment, and 1.66 in the YC treatment. The reduction between control and probiotic and control and YC was significant (p=0.021).

**Conclusion:**

This study demonstrates the ability of the novel probiotic and the YC to reduce the load of SE in layer ceca.

## Introduction

*Salmonella* is a significant cause of foodborne illness worldwide; in the United States, an estimate of 18.3 cases/100,000 population is reported [1]. The EU 20.4 cases/100,000 population are observed [2]. *Salmonella* Enteritidis (SE) is one of the most commonly isolated serotypes from clinical cases of salmonellosis, accounting for 14.5% of the US cases in 2012 and 39.5% of European cases in 2013 [2,3]. Approximately 29% of human salmonellosis cases can be attributed to poultry products [4]. This is not surprising as it is generally accepted that many serovars of *Salmonella* *enterica* can thrive in the gastrointestinal tract of poultry.

The poultry industry strives to reduce the risk of *Salmonella* in processing and production, including but not limited to processing aids, washing, biosecurity, vaccines, and a plethora of non-antimicrobial feed additives.

The market is filled with many different types of feed additives that are related with different modes of action aiming to improve health and performance of all agricultural species, including poultry [5,6]. *Saccharomyces cerevisiae* yeast cell wall (YCW) has been shown to improve intestinal health in *Clostridium* challenged broilers [7] and nutritionally challenged broilers [8]. YCW can improve economic performance and egg quality parameters when it is included in the diets of laying hens [9,10] and a reduction of *Salmonella* Typhimurium levels [11] and SE [12] in layer ceca is observed. YCW has also been shown to reduce the levels of *Salmonella* Heidelberg in broilers [13]. Mannanoligosaccharide supplementation resulted in reduced SE shedding from broiler chickens [14]. Many different species and strains of probiotic bacteria have been commercialized for the poultry industry. *Bacillus subtilis* probiotics have been shown to improve broiler performance and intestinal microbiota [15] and improve performance in birds with necrotic enteritis [16]. *Bacillus amyloliquefaciens* has been shown to improve feed conversion ratio in broilers [17]. *Bacillus subtilis* and *Bacillus licheniformis* probiotics have been shown to improve performance and reduce *Escherichia coli* shedding in laying hens [18]. There are also data to support the reduction of *Salmonella* in broilers with a *B. subtilis* probiotic [19]. Data related to *Salmonella* reduction with *Bacillus* probiotics in laying hens are limited. *Bacillus cereus* var. *toyoi* reduced the prevalence of SE in layer ceca [20]. Another commonly used feed additive is the yeast culture (YC), it has been shown to have performance and immunomodulatory effects in broilers [21].

This study aimed to address the ability of a commercially available YCW, a novel probiotic blend, or a commercially available YC to reduce the level of SE in the ceca of laying type pullets.

## Materials and Methods

### Ethical approval

The US National Research Council’s guidelines for the care and use of laboratory animals were followed.

### Animal husbandry and diets

Sixty Hy-Line Brown pullets were obtained from a commercial pullet rearing facility at approximately 6 weeks of age. The birds were transported to the research site and raised together for 3 weeks on control mash feed. The birds were then randomly assigned to 15 replicate blocks of four pens each. The pens provided 0.14 m^2^/bird. The birds had access to mash feed and water *ad libitum* for the duration of the trial. A basal diet was formulated to meet or exceed the current United States National Research Council recommendations for the age of bird. The basal diet was divided into four equal portions. All birds were individually weighed at the start of the trial (day 0) and at trial termination (day 10). The treatments were as follows: Without supplementation (control) and with dietary supplementation of a commercially available YCW at 454 g/ton, or a commercially available probiotic containing *B. amyloliquefaciens*, *B. licheniformis*, and *Bacillus pumilus* (probiotic) at 454 g/ton, or a commercially available YC at 1133 g/ton. The pullets were fed with the experimental diets for a 3-day acclimation period. The inclusion rates were selected from the product labels.

### Inoculation and sample collection

After the acclimation, the birds were orally gavaged with 3×10^7^ CFU of a nalidixic acid-resistant SE. On 7 days post-challenge, all pullets were humanely euthanized. Ceca were aseptically removed and placed into sterile plastic bags for *Salmonella* isolation. Samples were packed on ice and shipped overnight to the laboratory for *Salmonella* analysis.

### Microbiology

For isolation and identification of *Salmonella*, individual cecal samples were weighed, and 50 ml of Tetrathionate Broth was added to each ceca sample. Samples were mixed using a stomacher for 1 min and incubated for 24 h at 42°C. A loop full of incubated media was struck to xylose lysine tergitol-4 (XLT-4) plates containing 25 μg of nalidixic acid/mL to facilitate the selection of the antimicrobial-resistant challenge organisms, and plates incubated for 24 h at 37°C. Suspect *Salmonella* colonies were confirmed and serogrouped using poly-O *Salmonella*-specific antiserum. *Salmonella* were enumerated using a modification of the most probable number (MPN) method [22]. A 1 mL sample of pre-incubation 1:10 Tetrathionate Broth from each sample was transferred to three adjacent wells in the first row of a 96-well 2 mL deep block. A 0.1 mL aliquot was transferred to 0.9 mL of Tetrathionate Broth in the second row. This process was repeated for remaining rows producing 5, 10-fold dilutions. Blocks were incubated for 24 h at 42°C. One microliter of each well was transferred onto XLT-4 agar containing nalidixic acid with a sterile multichannel pipette and plates were incubated for 24 h at 37°C. The final dilution of each sample was recorded, and MPN calculations were performed as previously described. Suspect *Salmonella* isolates were confirmed by poly-O antisera.

### Statistical analysis

The lower quantitative limit of the MPN was 0.3×10^0^ MPN/mL, and the upper quantitative limit was 1.1×10^5^ MPN/mL. *Salmonella* prevalences in ceca samples were compared among the treatment groups using Fisher’s exact test. *Salmonella* MPNs in culture-positive ceca were compared among groups using the Kruskal–Wallis test, with *post hoc* pairwise comparisons performed using Dunn’s procedure. For the comparison of *Salmonella* MPNs, samples with a negative culture result by the MPN method, but a positive result by primary or secondary enrichment was arbitrarily assigned an MPN equal to one-half of the minimum detection limit of the MPN assay. MPNs were log transformed before statistical analysis. All statistical testing assumed a two-sided alternative hypothesis and p<0.05 was considered statistically significant. Analyses were performed using commercially available statistical software (Stata version 15.1, StataCorp LLC, College Station, TX, USA).

## Results and Discussion

There were neither significant differences in body weight nor weight gain among treatments. This finding was expected due to the short duration of the trial. *Salmonella* prevalences in ceca are presented in Table-1. The untreated control and YC groups had 14/15 birds or 93.3% test positive for *Salmonella*. The YCW and probiotic treatment groups had 15/15 or 100% of the ceca test positive for *Salmonella*. There was no significant difference among treatments with respect to *Salmonella* prevalence in ceca samples (p=1.00). All *Salmonella* isolates obtained from ceca were identified as belonging to serogroup D, which was consistent with the SE challenge strain. *Salmonella* MPNs for culture-positive ceca samples are shown in Table-2. There was a significant difference among treatments (p=0.021). The untreated control group and the YCW group were not significantly different from each other, but both had a higher median *Salmonella* log_10_ MPN/g than the probiotic and YC groups. The values for probiotic and YC groups were not significantly different from each other. The probiotic group had a mean reduction of 0.79 log_10_ MPN/g compared to the control; the YC had a similar mean reduction of 0.86 log_10_ MPN/g. The YC group had numerically the lowest MPN/g of *Salmonella* in the cecal contents, but the largest standard deviation compared to the other groups. Although the previous work has shown the ability of YCW [11,13] and mannooligosaccharide supplementation [14] to reduce *Salmonella* numbers or prevalence in chicken intestines, these results were not confirmed in this experiment. Variable results in reducing *Salmonella* are also reported for YCW treatments [23]. The probiotic group had a similar reduction in *Salmonella* numbers to the YC and a smaller standard deviation. YC has reduced the level of *S. Typhimurium* using an *in vitro* model [24]. This study also showed an increase in acetate and butyrate production in the ceca fermentation. Organic acids can reduce *Salmonella* in poultry [25]. The reduction of SE in poultry by YC has been discussed in popular press, but the authors found no peer-reviewed studies to support this. A study by Kiros *et al*. showed no reduction of *Salmonella* Heidelberg in broiler ceca by YC [13]. The reduction of *Salmonella* in poultry by *Bacillus* species in poultry has been demonstrated previously [20,26]. Probiotics can positively impact the intestinal microflora of chickens [27]. *B. amyloliquefaciens* has reduced the level of *E. coli* in broiler chicken digesta [28]. The reduction of SE could be attributed to a modification of the microflora in the ceca.

**Table-1:** *Salmonella* prevalence (%) in ceca samples by treatment group. Fifteen individually housed birds were sampled from each treatment group at 7 days post-challenge.

Treatment	Number of samples	Number of positive (%)	^†^p-value
1 – Control	15	14 (93.3)^a^	1.00
2 – YCW (454 g/t)	15	15 (100)^a^	
3 – Probiotic (454 g/t)	15	15 (100)^a^	
4 – YC (1133 g/t)	15	14 (93.3)^a^	
Total	60	58 (96.7)	

† Fisher’s exact test.

a Percentages with a superscript in common do not differ with a level of significance of 5% when using the Bonferroni multiple comparison procedure

**Table-2:** Summary of *Salmonella* log_10_ most probable number/g in culture-positive ceca samples by treatment. Fifteen individually housed birds were sampled from each treatment group at 7 days post-challenge.

Treatment	n	Mean (SD)	Median	^†^p-value
1 – Control	14	2.52 (0.98)	2.36^a^	0.021
2 – YCW (454 g/t)	15	2.49 (0.66)	2.44^a^	
3 – Probiotic (454 g/t)	15	1.73 (0.85)	1.43^b^	
4 – YC (1133 g/t)	14	1.66 (1.23)	1.37^b^	

† Kruskal–Wallis test.

a,b Medians with a superscript in common do not differ with a level of significance of 5% when using Dunn’s multiple comparison procedure

## Conclusion

The uniform reduction of SE in the ceca in the probiotic treatment group demonstrates that this product is a possible pre-harvest food safety intervention by reducing a load of *Salmonella* in the ceca, thus reducing the possible contamination to the environment and consequently the eggshell. The YC treatment also reduced the average *Salmonella* load in the ceca. Both YC and probiotic should be further investigated to better understand their impacts on *Salmonella* in poultry.

## Authors’ Contributions

PTP, TAG, MDS, and CLH planned and designed the study. MDS, CLH, and VB collected and analyzed data. PTP, TAG, and RDB analyzed data and contributed to the manuscript. All authors read and approved the final manuscript.
